# Expression and correlation of SOCS3 and Eotaxin mRNA and proteins levels in nasal mucosal tissue of allergic rhinitis patients

**DOI:** 10.3389/fimmu.2025.1561650

**Published:** 2025-06-11

**Authors:** Zhaopeng Kang, Lian Xu, Ping Ai, Wei Qu, Tang Li, Lixin Wang

**Affiliations:** ^1^ Department of Andrology, Renmin Hospital, Hubei University of Medicine, Shiyan, Hubei, China; ^2^ Department of Otolaryngology Head and Neck Surgery, Renmin Hospital, Hubei University of Medicine, Shiyan, Hubei, China

**Keywords:** suppressor of cytokine signaling 3, eosinophil, eosinophil chemotactic factor, allergic rhinitis, expression

## Abstract

**Objective:**

This study aims to investigate the expression and interaction mechanisms of Suppressor of Cytokine Signaling 3 (SOCS3) and Eotaxin in the nasal mucosal tissues of patients with Allergic Rhinitis (AR).

**Methods:**

In this retrospective study, we selected nasal mucosa tissues from 35 AR patients as the AR group, and nasal mucosa tissues from 22 patients with isolated nasal septum deviation as the control group. Utilizing reverse transcription polymerase chain reaction (RT-PCR) techniques, we measured the expression levels of SOCS3 mRNA and Eotaxin mRNA in both groups. Additionally, immunohistochemical methods were employed to assess the protein expression of SOCS3 and Eotaxin in these tissues.

**Results:**

The average optical density value of SOCS3 protein in the AR group was 0.270 ± 0.05, significantly higher than 0.160 ± 0.04 in the control group (P<0.01); the average optical density value of Eotaxin protein in the AR group was 0.240 ± 0.04, also significantly higher than 0.164 ± 0.03 in the control group (P<0.01). At the mRNA level, the ratio of SOCS3 mRNA to GAPDH in the AR group was 0.83 ± 0.27, and the ratio of Eotaxin mRNA to GAPDH was 0.71 ± 0.21, both significantly higher than 0.32 ± 0.11 and 0.22 ± 0.08 in the control group. Besides, the expression levels of SOCS3 protein and mRNA in the nasal mucosal tissues of AR patients were positively correlated with the expression of Eotaxin protein and mRNA (r=0.927, P<0.01; r=0.854, P<0.01).

**Conclusion:**

The high expression of SOCS3 and Eotaxin in the nasal mucosal tissues of AR, as well as the positive correlation between them, suggest that they may play an important role in the pathogenesis of AR. This study provides new experimental evidence for in-depth understanding of the pathogenesis of AR and new potential targets for the treatment of AR.

## 1 Introduction

Allergic rhinitis (AR) is a common allergic disease. Its clinical manifestations mainly include symptoms such as nasal congestion, runny nose, sneezing and nasal itching, and the incidence rate is increasing year by year (1, 2). The occurrence of AR is closely related to genetic factors, environmental factors and abnormal responses of the immune system, among which IgE-mediated type I hypersensitivity reaction is the main one (3, 4). Eosinophil chemotactic factor (Eotaxin) plays a key role in allergic diseases, especially AR. It participates in the local inflammatory reaction process by activating and attracting eosinophils to migrate to the inflammatory site (5). Existing studies have shown that the expression level of Eotaxin protein in the nasal mucosal tissues of AR patients is significantly increased, and the number of positive expression cells of this protein is positively correlated with the number of eosinophils (4).

On the other hand, suppressor of cytokine signaling-3 (SOCS3), as a newly discovered negative feedback regulatory molecule, mainly acts on the JAK-STAT signaling pathway and can effectively inhibit the signal transduction of various cytokines such as IL-4, IL-5, IL-13, etc., thereby exerting an inhibitory effect on the Th2-type immune response (6). SOCS3 not only plays an important role in maintaining immune balance under normal physiological conditions but also has been confirmed to have anti-inflammatory effects in many disease models (7–9). It is worth noting that although SOCS3 is considered to be mainly expressed in Th2 cells and can inhibit Th1-type immune responses, its specific functions in allergic diseases, especially AR, are not completely clear. Considering that SOCS3 may affect the process of AR through the regulation of Th2 cell activity and its related cytokines, it is necessary to deeply explore the interaction between SOCS3 and Eotaxin and its potential significance in the pathogenesis of AR. SOCS3 can also indirectly influence the expression of Eotaxin via relevant pathways. While Eotaxin, as a chemokine, not only directly recruits eosinophils but also activates Th2 cells to further promote the release of cytokines such as IL-4. This bidirectional regulation may form a positive feedback loop, exacerbating the Th2-type inflammatory response in AR. In addition, SOCS3 and Eotaxin proteins are expressed in nasal mucosal epithelial cells, glandular cells, and vascular endothelial cells, mainly in monocytes. Therefore, exploring the co-expression pattern and interaction of SOCS3 and Eotaxin in nasal mucosal tissues is of great significance for elucidating the pathogenesis of AR.

At present, there are relatively few studies on the co-expression pattern of SOCS3 and Eotaxin in the nasal mucosal tissues of AR patients, and whether there is a direct or indirect connection between the two still needs further verification. In addition, previous studies have mostly focused on the functional exploration at the single molecule level, lacking studies that comprehensively analyze the interactions between different molecules from an overall perspective. This project aims to systematically evaluate the regulatory roles of SOCS3 in Th2-driven inflammation via JAK-STAT pathway suppression and the chemotactic function of Eotaxin in eosinophil recruitment in the pathological process of AR by detecting the expression of SOCS3 and Eotaxin proteins and their mRNAs in the nasal mucosal tissues of AR patients, combined with immunohistochemical techniques and RT-PCR techniques, and attempts to reveal their potential synergistic interaction in amplifying allergic inflammation. This study attempts to reveal a possible correlation between the two factors, we hope to provide a new perspective for understanding the complex pathological mechanism of AR through this study, and at the same time lay a theoretical foundation for the future development of treatment strategies targeting specific targets.

## 2 Materials and methods

### 2.1 Case collection

In this retrospective study, 35 AR patients who were treated in the outpatient or inpatient department from September 2023 to February 2024 were included, and the inferior turbinate mucosal tissues of 22 patients who underwent septoplasty during the same period were collected via endoscopic-guided dissection (ensuring minimal mechanical trauma) and selected as the control group. The inclusion criteria for patients were: (1) aged 18–60 years; (2) in line with the standard diagnosis of allergic rhinitis (10). The exclusion criteria included (1) family history of nasal polyps; (2) history of bronchial asthma; (3) patients intolerant to aspirin treatment.

This study has been approved by the hospital ethics committee of Renmin Hospital, Hubei University of Medicine (No. B24038) and follows the principles of the Helsinki Declaration. All participants signed the informed consent form before the operation.

### 2.2 Experimental reagents and materials

The rabbit anti-human SOCS3 antibody (catalog No. A01234, GenScript, Nanjing, China) was purchased from GenScript Company, located in Nanjing, China, and the rabbit anti-human Eotaxin protein antibody (catalog No. sc-56890, Santa Cruz Biotechnology, Dallas, TX, USA) was purchased from Santa Cruz Company in Dallas, TX, USA. The secondary antibody (goat anti-rabbit IgG, catalog No. BA1054, Wuhan Boster Biological Technology Co., Ltd., Wuhan, China) and 3,3’-Diaminobenzidine (DAB) chromogenic kit (catalog No. AR1022, Wuhan Boster Biological Technology Co., Ltd., Wuhan, China) were both obtained from Wuhan Boster Biological Technology Co., Ltd. in Wuhan, China. The upstream and downstream primers for SOCS3 and Eotaxin were synthesized by Shanghai Biotechnology Co., Ltd. in Shanghai, China, and the DNA marker was purchased from Beijing Dingguo Biological Technology Co., Ltd. in Beijing, China. In addition, absolute alcohol, hydrochloric acid (37% concentration), paraformaldehyde (4% concentration), and xylene were sourced from Wuhan Huisheng Biological Technology Co., Ltd. All reagents were of analytical purity and were used in accordance with the instructions provided by the manufacturer.

### 2.3 Detection of SOCS3 and Eotaxin protein expression

When detecting the expression of SOCS3 and Eotaxin proteins by immunohistochemistry, first, the nasal mucosal tissues of the two groups were completely immersed in a 4% paraformaldehyde solution for 24 hours, and then tissue embedding and sectioning were performed. After sectioning, routine dewaxing (sequential incubation in xylene for 10 min × 2, followed by rehydration through graded ethanol: 100%, 95%, 85%, 75%, 5 min each, and distilled water) was carried out; endogenous peroxidase was removed by heating, and then blocked with normal goat serum (1:10). Then add the primary antibody (rabbit anti-human SOCS3 antibody, titer 1:100; rabbit anti-human Eotaxin protein antibody, titer 1:100), and act at 37°C for 30 minutes. Then, after washing the slices with 0.01 mol/L PBS solution, add the secondary antibody (goat anti-rabbit IgG, titer 1:100), and act at 37°C for 30 minutes. Then add the SP complex (streptavidin-peroxidase complex, used for signal amplification) and act at 37°C for 30 minutes again. Finally, add the DAB solution for color development reaction for 5 minutes. After transparency treatment (incubation in xylene for 5 min × 2) and dehydration, the slices were sealed with neutral gum. Five fields were randomly selected from each slice under a 400× microscope, and the positive area was corrected for optical density, selected and filtered using Image-pro Plus 6.0 professional image analysis software, and the average optical density value was calculated.

### 2.4 Detection of SOCS3 mRNA and Eotaxin mRNA expression by RT-PCR

Total RNA was extracted from the nasal mucosal tissues using the Trizol method, following the manufacturer’s guidelines (11). The concentration and purity of RNA were determined by ultraviolet spectrophotometer to ensure that the A260/A280 ratio was between 1.8 - 2.0. 5 μg of total RNA was taken and cDNA was synthesized by reverse transcriptase (SuperScript III Reverse Transcriptase, Invitrogen). The upstream and downstream primer sequences of SOCS3 and Eotaxin were designed according to GeneBank. The information of primer sequences including upstream, downstream primer and the length of the amplified product was shown in the **Supplementary Table**. The PCR amplification conditions were as follows: pre-denaturation at 94°C for 10 minutes; denaturation at 94°C for 40 seconds, annealing at 56°C (GAPDH) or 54°C (SOCS3, Eotaxin) for 40 seconds, extension at 72°C for 40 seconds, for a total of 30 cycles; finally, extension at 72°C for 10 minutes. The amplified products were detected by 1.5% agarose gel electrophoresis, and the results were photographed and recorded using the Gel Doc system.

### 2.5 Statistical processing methods

The experimental data were statistically analyzed using SPSS 13.0 statistical software. The comparison of the expression levels of SOCS3 and Eotaxin proteins and their mRNAs was performed using the independent sample t-test. Correlation analysis was performed using the Pearson correlation coefficient. All data were subjected to the Shapiro-Wilk normality test, and parametric tests were performed only for data that met the normal distribution. P < 0.05 was considered statistically significant.

## 3 Results

### 3.1 General situation of patients

In this study, 35 patients diagnosed with allergic rhinitis (AR) were included, comprising 15 males and 20 females, with an age range of 28 to 45 years and a mean age of (37.8 ± 6.7) years. The control group consisted of 22 patients undergoing septoplasty, including 12 males and 10 females, with an average age of (38.5 ± 8.2) years.

Intergroup comparisons revealed significant differences in various demographic and clinical characteristics (see **Table 1**). A higher proportion of AR patients had atopic conditions (65% vs. 10%, p < 0.01) and a family history of allergies (70% vs. 15%, p < 0.01). Notably, none of the AR patients reported chronic respiratory conditions, while 22.7% of the control group did (p = 0.10). Regarding lifestyle factors, 22.9% of AR patients were current smokers compared to 13.6% in the control group (p = 0.25). Additionally, occupational exposure to allergens was reported by 30% of AR patients versus only 5% of controls (p < 0.05).

**Table 1:** Baseline data of included patients.

Characteristic	Allergic Rhinitis Group (n = 35)	Control Group (n = 22)	p-value
Gender (M/F)	15/20	12/10	0.54
Age (years)	37.8 ± 6.7	38.5 ± 8.2	0.67
Atopic Conditions	65% (n = 23)	10% (n = 2)	< 0.01
Family History of Allergies	70% (n = 24)	15% (n = 3)	< 0.01
Chronic Respiratory Conditions	0% (n = 0)	22.7% (n = 5)	0.10
Current Smokers	22.9% (n = 8)	13.6% (n = 3)	0.25
Occupational Allergen Exposure	30% (n = 10)	5% (n = 1)	< 0.05
History of Allergic Reactions	75% (n = 26)	0% (n = 0)	< 0.001
Antihistamine Use	80% (n = 28)	5% (n = 1)	< 0.001
Intranasal Corticosteroids Use	60% (n = 21)	0% (n = 0)	< 0.001
Average Total Symptom Score	8.2 ± 2.1	1.5 ± 0.9	< 0.001

Furthermore, a significant number of AR patients had a history of allergic reactions (75% vs. 0%, p < 0.001) and reported higher usage rates of antihistamines (80% vs. 5%, p < 0.001) and intranasal corticosteroids (60% vs. 0%, p < 0.001). The average total symptom score was markedly higher in the AR group (8.2 ± 2.1) compared to the control group (1.5 ± 0.9, p < 0.001). These findings highlight the distinct characteristics of the AR patients in comparison to the control group, which may influence the study outcomes (**Table 1**).

### 3.2 Comparison of SOCS3 and Eotaxin protein expression levels

**Figure 1:**
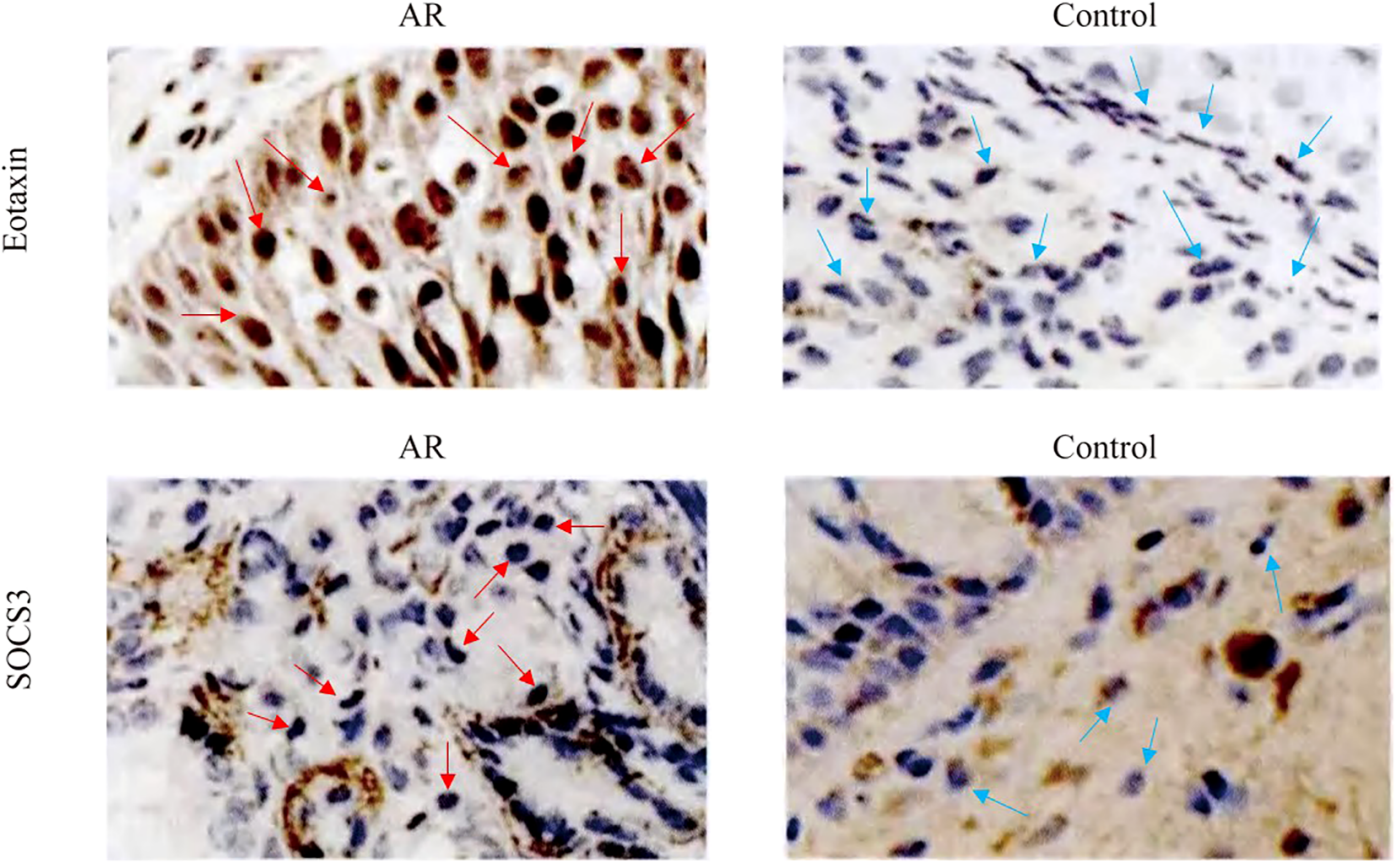
Expression of SOCS3 and Eotaxin proteins in the AR group and the control group detected by immunohistochemical method.

The expression levels of SOCS3 (0.27 ± 0.05) and Eotaxin proteins (0.24 ± 0.04) in the nasal mucosal tissues of the AR group were significantly higher than those in the control group (SOCS3, 0.16 ± 0.04; Eotaxin protein, 0.164 ± 0.03) (P < 0.01) (**Table 2**). It can be seen that the cytoplasm staining in the AR group is more obvious, showing brown-yellow or brownish particles (**Figure 1**).

**Table 2:** Comparison of protein expression levels of SOCS3 and Eotaxin in the two groups.

Protein	The AR group (n=24)	The control group (n=22)	*t*	*P*
SOCS3	0.27±0.05	0.16±0.04	8.027	<0.01
Eotaxin protein	0.24±0.04	0.164±0.03	8.185	<0.01

AR, allergic rhinitis; SOCS3, suppressor of cytokime singnaling-3.

### 3.3 Correlation analysis of SOCS3 and Eotaxin protein expression levels in the AR group

**Figure 2:**
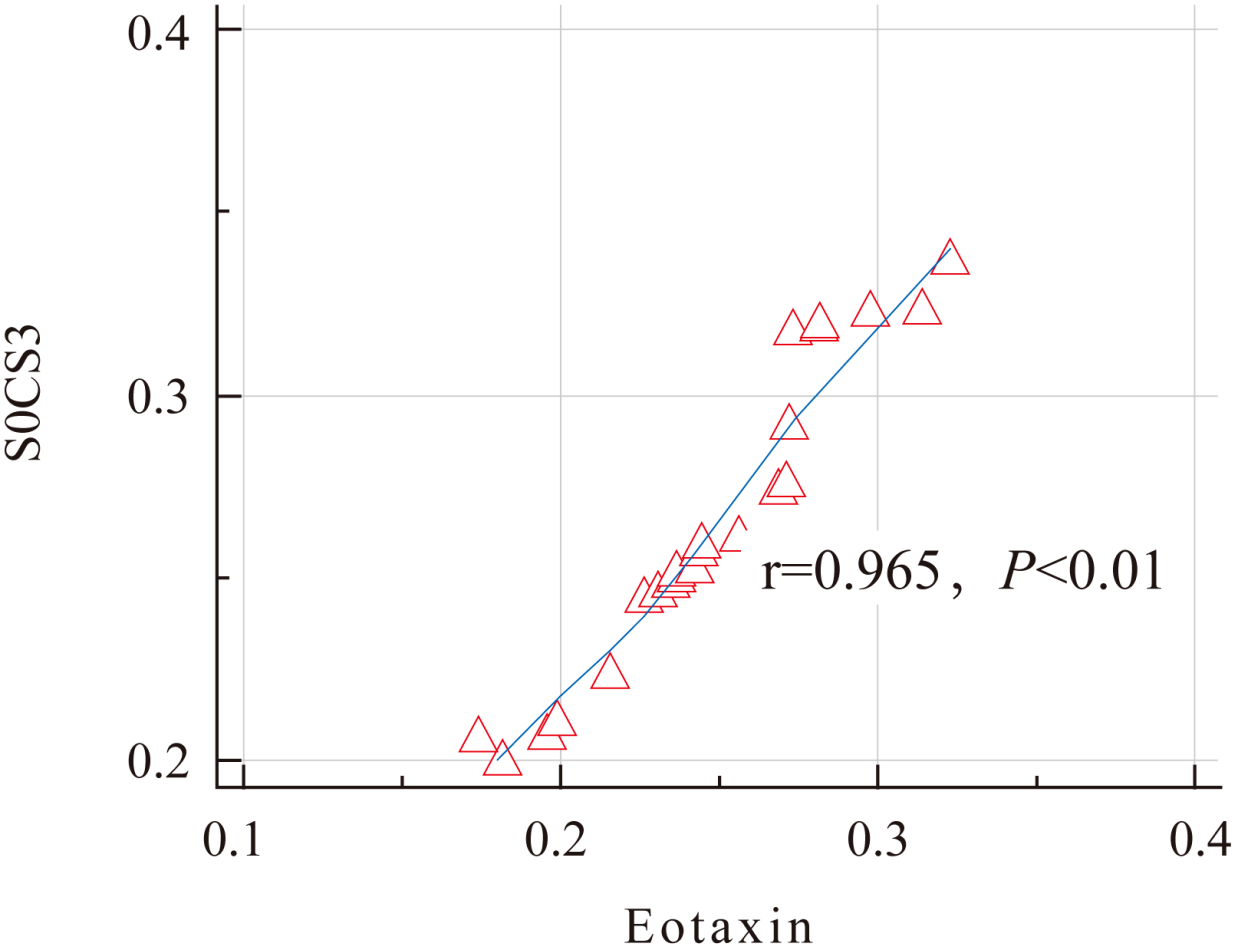
Correlation analysis of the expression levels of SOCS3 and Eotaxin proteins in the AR group.

Further analysis found that the expression levels of SOCS3 and Eotaxin proteins in the nasal mucosal tissues of AR patients were positively correlated (r = 0.927, P < 0.01) (**Figure 2**). This result indicates that in the nasal mucosal tissues of AR patients, the expressions of SOCS3 and Eotaxin proteins may have some kind of interaction or common regulatory mechanism.

### 3.4 Comparison of SOCS3 mRNA and Eotaxin mRNA expression levels

**Figure 3:**
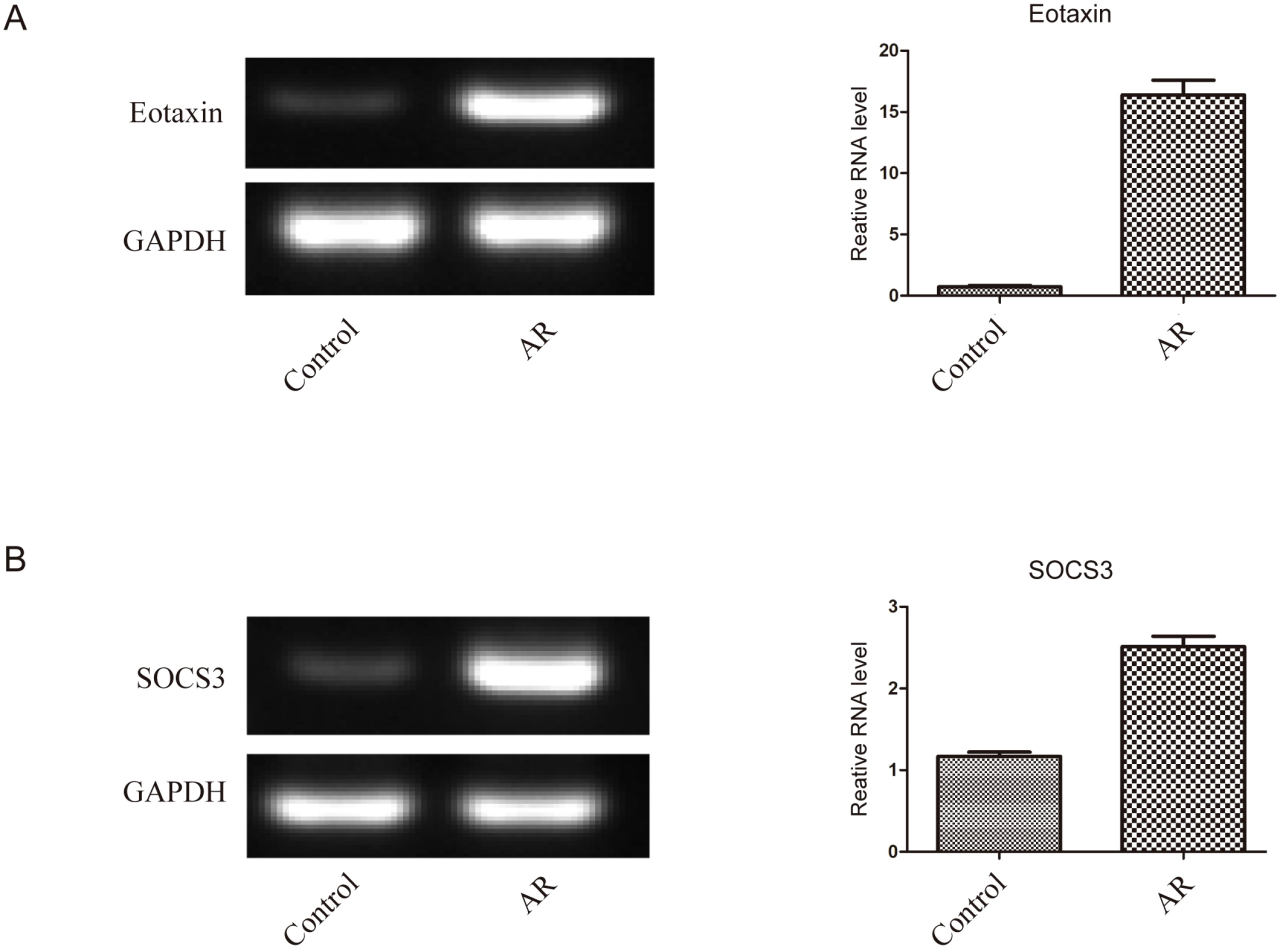
The expression of SOCS3 mRNA and Eotaxin mRNA in nasal mucosal tissues of the two groups of patients was detected by RT-PCR **(A)** Expression level of Eotaxin mRNA, **(B)** Expression level of SOCS3 mRNA.

**Figure 4:**
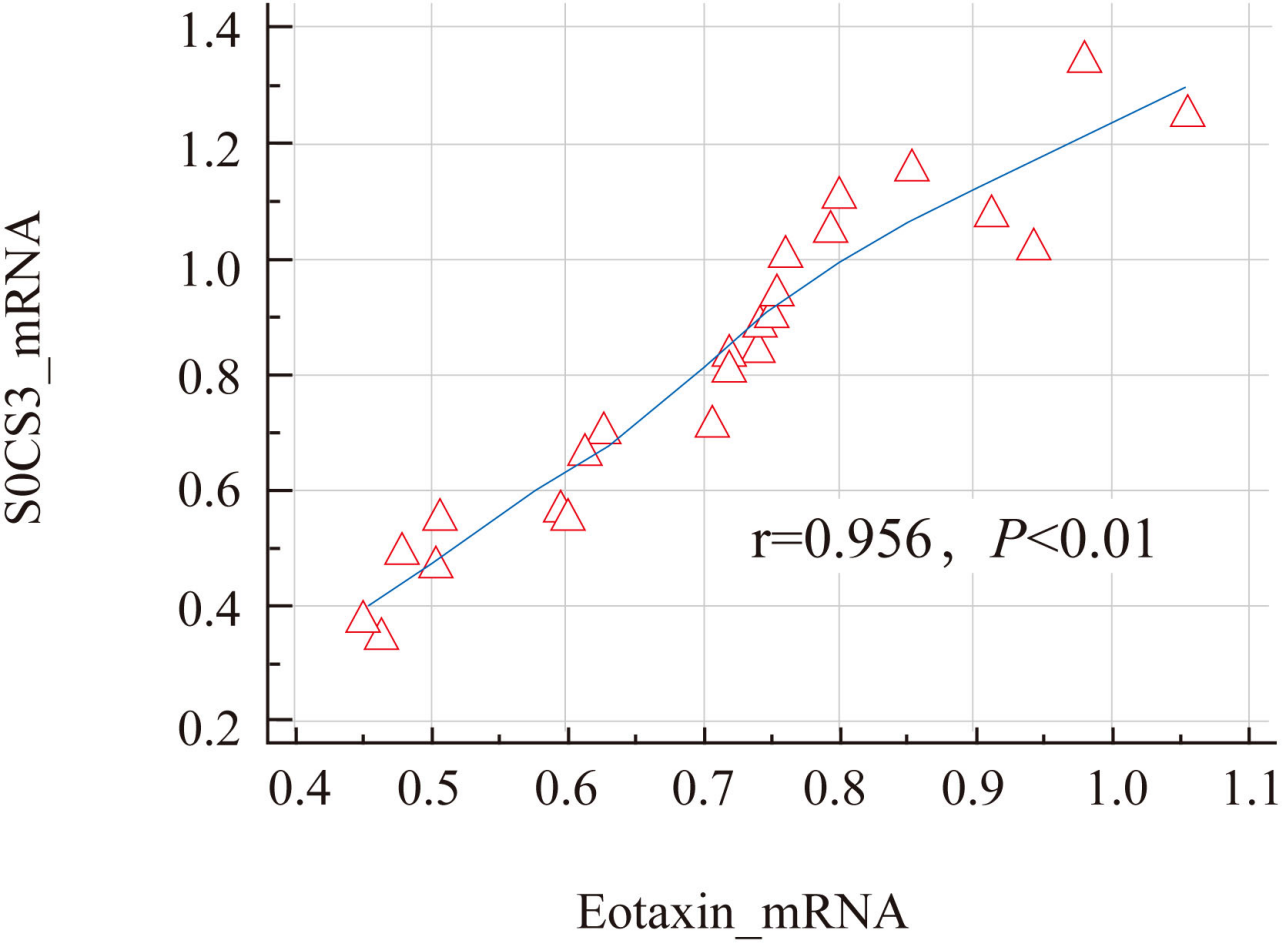
Correlation analysis of SOCS3 mRNA and Eotaxin mRNA in nasal mucosal tissues of AR patients.

The results showed that the expression levels of SOCS3 mRNA (0.83 ± 0.27) and Eotaxin mRNA (0.71 ± 0.21) in the nasal mucosal tissues of the AR group were significantly higher than those of the control group (SOCS3, 0.32 ± 0.11; Eotaxin, 0.22 ± 0.08) (P < 0.01). The specific data are shown in **Table 3**; **Figure 3**. In addition, the expression levels of SOCS3 mRNA and Eotaxin mRNA in the nasal mucosal tissues of AR patients were also positively correlated (r = 0.854, P < 0.01) (**Figure 4**).

**Table 3:** Comparison of expression levels of SOCS3 mRNA and Eotaxin mRNA.

mRNA	The AR group (n=24)	The control group (n=22)	t	P
SOCS3/GAPDH	0.83±0.27	0.32±0.11	9.245	<0.01
Eotaxin/GAPDH	0.71±0.21	0.22±0.08	9.731	<0.01

AR, allergic rhinitis; SOCS3, suppressor of cytokime singnaling-3.

## 4 Discussion

Unlike previous studies that primarily focused on isolated analyses of SOCS3 or Eotaxin in AR (4, 6), this study was the first to systematically investigate their co-expression patterns and interactions in nasal mucosal tissues. Using combined immunohistochemical and RT-PCR approaches, we demonstrated that both SOCS3 and Eotaxin proteins and mRNAs were significantly upregulated in AR patients compared to controls, with a strong positive correlation between their expression levels. This dual-molecule analysis reveals a previously unrecognized synergistic relationship between SOCS3 and Eotaxin, suggesting their collective role in amplifying Th2-driven inflammation in AR.

SOCS3 is an important negative regulatory molecule, which mainly regulates the signal transduction of various cytokines by inhibiting the JAK-STAT signaling pathway (12). In AR, the expression of SOCS3 mRNA and serum IL-4 levels were significantly increased, and there was a significant positive correlation between them (p < 0.05) (6).Th2 cells and their cytokines are dominant in the body of AR patients, and Th2 cells mainly synthesize and secrete a variety of inflammatory cytokines. SOCS3, predominantly expressed in Th2 cells under IL-4 stimulation, inhibits IL-12/STAT4 signaling pathways required for Th1 differentiation, thereby suppressing Th1 cytokine production and reinforcing Th2-mediated allergic responses. This mechanism does not directly repress Th2 cytokines but instead disrupts the counter-regulatory Th1 signals, allowing unchecked Th2 polarization. Such reciprocal inhibition between SOCS3 (Th2-associated) and SOCS5 (Th1-associated) underpins the Th1/Th2 imbalance observed in allergic diseases (13). Therefore, the high expression of SOCS3 may be one of the important reasons for the imbalance of Th1/Th2 ratio in AR patients.

The latest research shows that SOCS3 not only plays a key role in inhibiting the inflammatory response but also has an important role in maintaining immune balance (14). In allergic diseases, the upregulation of SOCS3 may be a self-protection mechanism of the body against excessive inflammatory responses. However, our findings raise a critical question: why does elevated SOCS3 fail to suppress Eotaxin-driven inflammation in AR? One plausible explanation is that SOCS3 may exhibit functional deficiency in this context. Post-translational modifications (e.g., phosphorylation) or competition with STAT6 for binding to cytokine receptors could impair its ability to inhibit IL-4/STAT6 signaling, thereby allowing sustained Eotaxin expression (14, 15). Alternatively, chronic Th2 inflammation might overwhelm SOCS3’s regulatory capacity, rendering it insufficient to break the positive feedback loop between Th2 cytokines and Eotaxin. This protective mechanism may not be sufficient to completely counteract the excessive activation of the Th2-type immune response, resulting in a persistent inflammatory state (15, 16). Our research results indicate that the expression of SOCS3 in the nasal mucosal tissues of AR patients is significantly increased, which may reflect the body’s attempt to inhibit the excessive Th2-type immune response by upregulating SOCS3, but fails to completely restore the immune balance.

Eotaxin is an important chemotactic factor that mainly attracts and activates eosinophils by binding to the CCR3 receptor and participates in local inflammatory responses (17).In the nasal mucosal tissues of patients with allergic rhinitis, the expression of Eotaxin is mainly concentrated around epithelial cells and inflammatory cells. Existing studies have shown that the expression of Eotaxin is significantly increased in the nasal mucosal tissues of AR patients, and the number of its positive expression cells is positively correlated with the number of eosinophils (18). Eotaxin can also induce the differentiation and proliferation of Th2 cells and promote the secretion of cytokines such as IL-4 and IL-5, further aggravating allergic symptoms (19). The latest research further reveals the multiple mechanism of action of Eotaxin in AR. In addition to directly attracting eosinophils, Eotaxin can also increase vascular permeability by activating vascular endothelial cells, thereby promoting the exudation of inflammatory mediators (20). In addition, Eotaxin can also induce the degranulation of mast cells, release histamine and other inflammatory mediators, and intensify the inflammatory response (21). At the cellular pathway level, as a CC chemokine, Eotaxin activates multiple signaling pathways by binding to the CCR3 receptor, including the Ras-Raf-MEK-ERK (MAPK) pathway and the PI3K/Akt pathway (22). The activation of these signaling pathways promotes the migration, proliferation and release of inflammatory mediators of eosinophils, leading to the intensification of the inflammatory response of allergic rhinitis. This study found that the expression levels of SOCS3 and Eotaxin are positively correlated, which may imply the existence of some kind of interaction or common regulatory mechanism between the two. To clarify, SOCS3 does not directly enhance Th2 activity but instead reinforces Th2 dominance by suppressing Th1 differentiation: induced by IL-4 in Th2 cells, SOCS3 inhibits IL-12/STAT4 signaling required for Th1 polarization, reducing Th1-derived counter-regulatory cytokines and allowing Th2 cells to predominate. This Th2-skewed environment may indirectly upregulate Eotaxin, a Th2-associated chemokine. A distinct possibility is a direct molecular interaction, where SOCS3 and Eotaxin form a feedback loop (e.g., Eotaxin-driven eosinophil recruitment may stimulate SOCS3 expression via cytokine feedback, while SOCS3 sustains Th2 activity to promote Eotaxin production). Future studies need to further explore the specific mechanism of this interaction to clarify the specific functions of SOCS3 and Eotaxin in AR.

Our discovery of a robust positive correlation between SOCS3 and Eotaxin (r=0.927 for proteins; r=0.854 for mRNAs) extends beyond prior reports of their individual upregulation in AR (6, 19). This finding implied a bidirectional regulatory loop—where SOCS3 may enhance Th2 cell activity to promote Eotaxin production, while Eotaxin-driven eosinophil recruitment could further stimulate SOCS3 expression via cytokine feedback. Such a mechanism, not previously described in AR, highlights the need for therapeutic strategies targeting both molecules to disrupt this pro-inflammatory axis. One possibility is that SOCS3 indirectly increases the expression level of Eotaxin by enhancing the activity of Th2 cells. Another possibility is that SOCS3 directly interacts with Eotaxin to form a feedback regulatory loop. Specifically, IL-4 can induce the expression of Eotaxin, while SOCS3 down-regulates the expression of Eotaxin by inhibiting the IL-4/STAT6 signaling pathway (13). Our research results support this hypothesis that in AR patients, the interaction between SOCS3 and Eotaxin may form a complex regulatory network and jointly participate in the pathological process of AR. Understanding the mechanism of action of SOCS3 and Eotaxin in AR is of great significance for the development of new treatment strategies. Due to the key role of Eotaxin in AR, treatment strategies targeting Eotaxin or its receptor CCR3 may become an important direction in the future.

Although this study provides some valuable findings, there are still some limitations. First, the sample size is relatively small, which may affect the universality and representativeness of the results. Larger-scale studies are needed in the future to verify these preliminary findings. Secondly, although we observed a positive correlation between the expression levels of SOCS3 and Eotaxin, the specific interaction mechanism is not completely clear. Importantly, our study did not address whether elevated SOCS3 itself contributes to unintended immunological consequences. For instance, SOCS3’s broad suppression of JAK-STAT pathways (e.g., STAT1/STAT3) could impair antiviral defenses or tissue repair mechanisms, potentially explaining the clinical association between AR and prolonged viral rhinitis (21). Furthermore, excessive SOCS3 activity might exacerbate Th2 polarization by suppressing Th1 counter-regulation, creating a self-reinforcing cycle of inflammation. Future studies can use more advanced molecular biology techniques, such as gene knockout or overexpression experiments, combined with pathway-specific inhibitors, to dissect SOCS3’s dual roles in AR. Additionally, single-cell sequencing could identify cell subtypes where SOCS3-Eotaxin co-expression drives pathology, guiding targeted therapies. Finally, the therapeutic implications of SOCS3 modulation warrant careful consideration. While targeting Eotaxin/CCR3 remains a promising strategy for AR (22), our data suggest that SOCS3 manipulation—whether through inhibition to restore immune balance or enhancement to suppress Th2 responses—requires precision. For example, small-molecule SOCS3 enhancers might be combined with CCR3 antagonists to simultaneously dampen eosinophil recruitment and Th2 cytokine production. Conversely, in patients with SOCS3 functional deficiency, gene editing to restore its activity could offer a novel approach. These strategies, however, must be rigorously tested in preclinical models to avoid unintended immunosuppression or exacerbation of inflammation.

## 5 Conclusion

In conclusion, this study revealed the high expression and positive correlation of SOCS3 and Eotaxin in the nasal mucosal tissues of AR, providing a new perspective for further understanding the pathogenesis of AR and laying a foundation for the development of treatment strategies targeting these molecules. Future studies need to verify these findings in a larger sample size and deeply explore the specific interaction mechanism between SOCS3 and Eotaxin, in order to provide more scientific basis for the treatment of AR.

## Data Availability

The original contributions presented in the study are included in the article/**Supplementary Material**. Further inquiries can be directed to the corresponding author.
