# Supplementary figures and images for: Expression and correlation of SOCS3 and Eotaxin mRNA and proteins levels in nasal mucosal tissue of allergic rhinitis patients
Source: Front Immunol. 2025 Jun 11;16:1561650. doi: 10.3389/fimmu.2025.1561650 (PMC12187767; doi:10.3389/fimmu.2025.1561650)

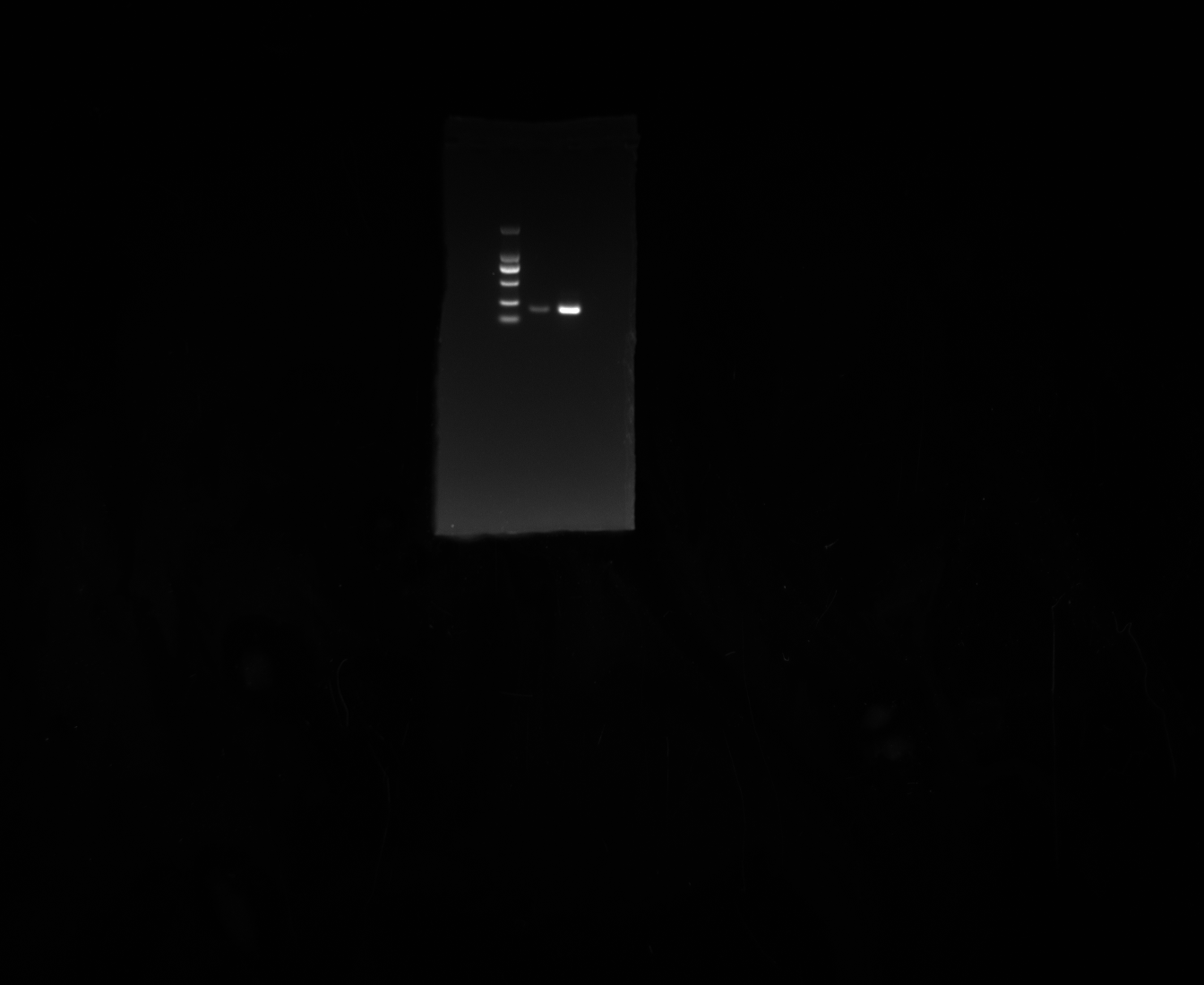

Supplement: Supplementary file 1 [file Image1.tif]

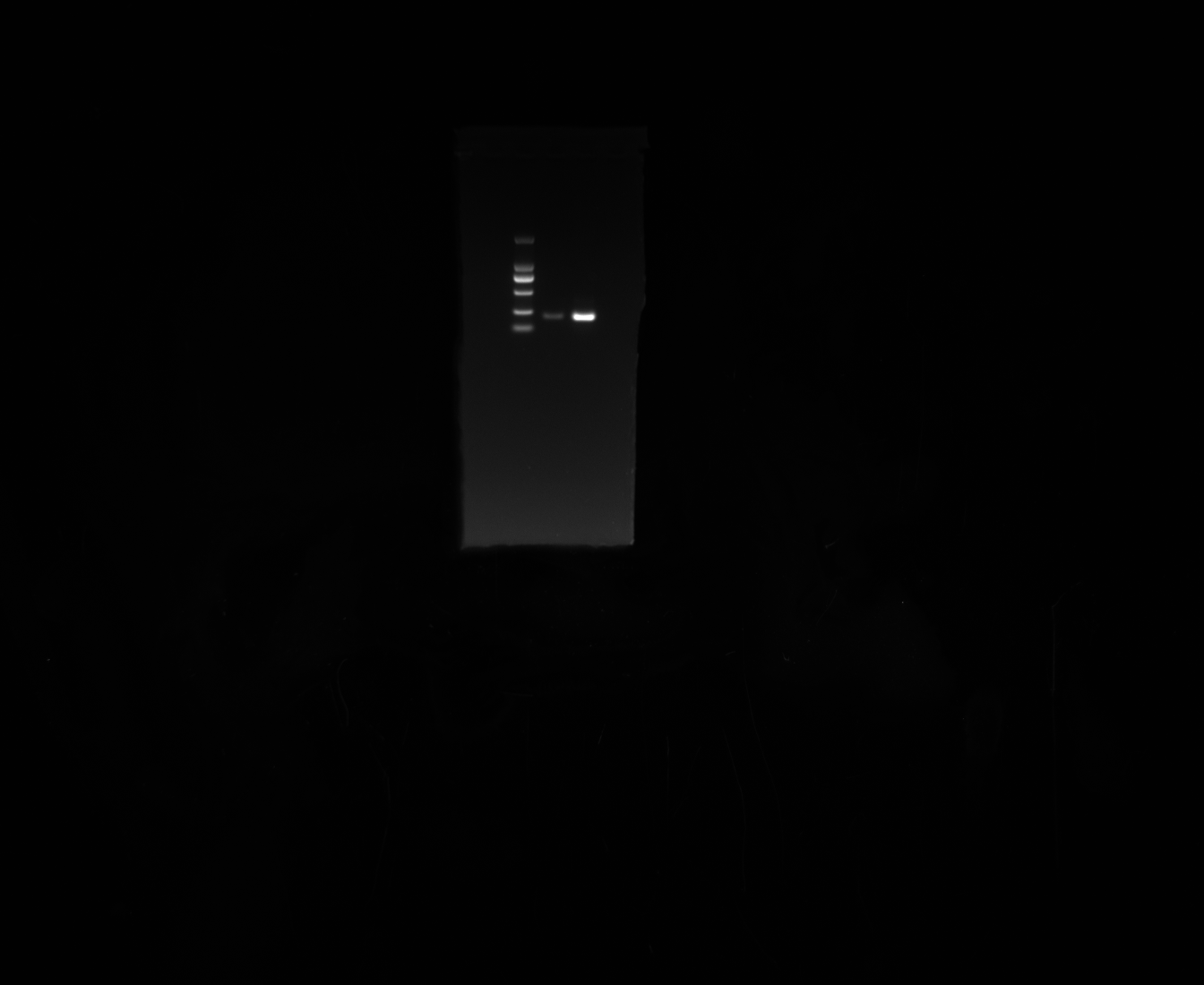

Supplement: Supplementary file 2 [file Image2.tif]

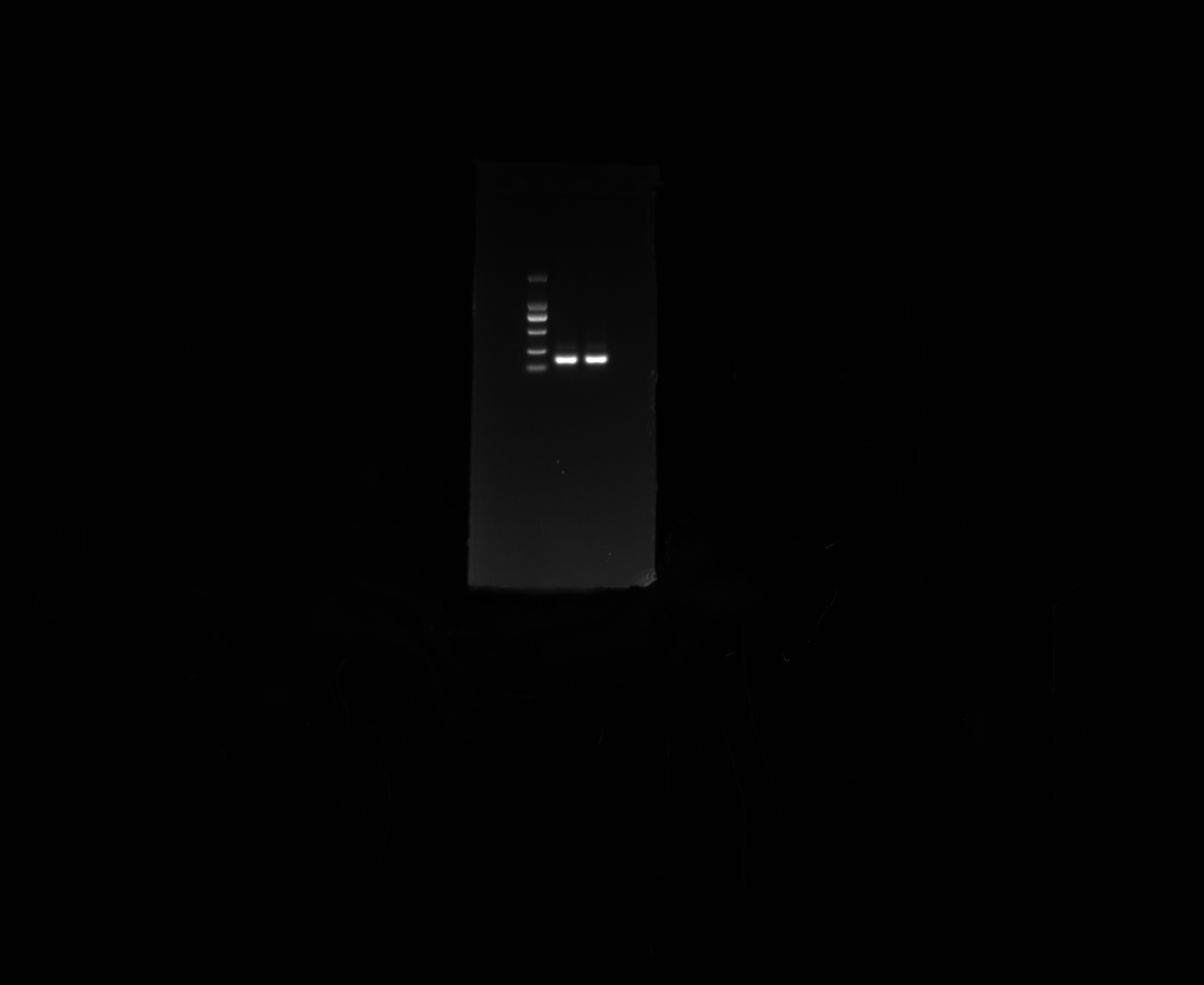

Supplement: Supplementary file 3 [file Image3.tif]

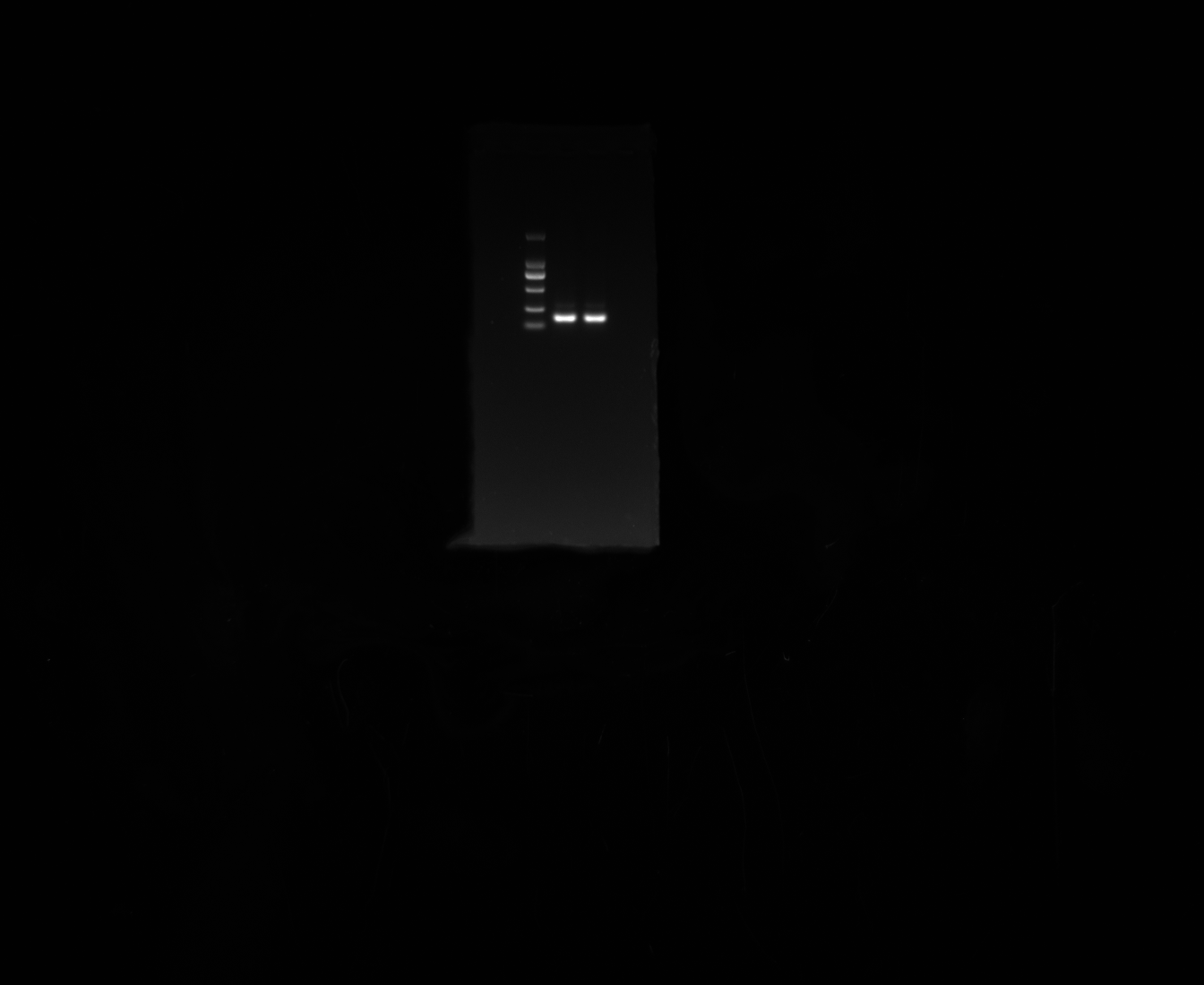

Supplement: Supplementary file 4 [file Image4.tif]
