# Supplementary material for: Expression and correlation of SOCS3 and Eotaxin mRNA and proteins levels in nasal mucosal tissue of allergic rhinitis patients
Source: Front Immunol. 2025 Jun 11;16:1561650. doi: 10.3389/fimmu.2025.1561650 (PMC12187767; doi:10.3389/fimmu.2025.1561650)
Supplement: Supplementary file 5 [file Table1.docx]

Supplementary Table The information of primer sequences

|  | **Upstream primer** | **Downstream primer** | **Length of the amplified product**  **(bp)** |
| --- | --- | --- | --- |
| **SOCS3** | 5'-CAGCTCCAAGAGCGAGTAC-3' | 5'-GTTCTTGGTCCCAGACT-3' | 225 |
| **Eotaxin** | 5'-CATGAAGGTCTCCGCAGCACTTCT-3' | 5'-CCAGATACTTCATGGAATCCTGC-3' | 266 |
| **Internal reference gene GAPDH** | 5'-ATCATGTTTGAGACCTTTCAACA-3' | 5'-CATCTCTTGCTCGAAGTCCA-3' | 318 |
